# Meat Reduction in 5 to 8 Years Old Children—A Survey to Investigate the Role of Parental Meat Attachment †

**DOI:** 10.3390/foods10081756

**Published:** 2021-07-29

**Authors:** Julia Erhardt, Annemarie Olsen

**Affiliations:** Department of Food Science, Faculty of Science, Section for Design and Consumer Behaviour, University of Copenhagen, Rolighedsvej 26, 1958 Frederiksberg C, Denmark; julia.erhardt@outlook.com

**Keywords:** meat-reduction, children, eating behaviour, sustainability, healthy, Germany, food choice, food preference

## Abstract

It is by now well established that a plant-based and meatless or reduced-meat diet is an important contribution to a sustainability and healthy diet. This work discusses important determinants for parents of implementing a meat reduced diet for their children. A questionnaire was conducted with 90 parents of children aged 5–8 years living in Germany, where they had to choose one out of three options of a dish, namely meaty, reduced meat and no meat, for their child. The results show that the parent’s attachment to meat and the associated attitudes and habits play a crucial role in their meal choice and therefore eating behaviour, including consumed amounts of meat, of their child. Moreover, perceived tastiness, healthiness and balanced serving style, as well as the child’s preferences influences the parent’s decision. The findings of this work provide valuable insights to the food industry and food producers, health professionals and public health, as it highlights the background, as well as some drivers and barriers for parents choosing a dish with less meat for their children.

## 1. Introduction

Worldwide, meat is an important part of the human diet. It has a meaning that goes beyond its nutritional properties: it stands for high social status, strength, masculinity, sexuality and power [1]. Compared to other food, meat ranks trans-culturally highest in people’s hierarchy of food valuation [2]. However, eating meat is usually not done out of rational and conscious thoughts. Even though the *Theory of Reasoned Action* [3] and the resultant *Theory of Planned Behaviour* [4] explain that behaviour is based on intentions—which are in turn based on values, attitudes, subjective norms and perceived behavioural control—it was shown that the link between actual food choice and intentions is contradicting and faint [5,6,7].

This means that there is more required than a rational consumer model to explain why we eat what we eat: our food choice is manifold and based on many interacting and mostly unconscious reasons, such as habits and past behaviour, as well as hedonic appreciation [5,8]. Eating habits, in turn, are the result of socio-cultural factors, such as family meals, traditional dishes and social circles [8]. In addition, food preferences are rooted in pre-natal and post-natal age, as well as in early childhood [9,10]. Parents have a key function when it comes to their children’s eating behaviour [11,12,13], e.g., by being a role model to shape attitudes towards certain food, as well as parental control of the kind of food that is eaten at home and offered to the child.

Today, there is an increasing call for consuming less (or even no) meat for a variety of reasons. According to the United Nations Organisation report *World Population Prospects 2019: Highlights*, today’s world population of 7.8 billion will have increased to 9.7 billion in 2050 [14] and these people have to be fed. However, with our current food system, which is focusing on meat production, we are putting this long-term goal of feeding everyone at risk. Meat production has a major impact on the environment—it leads to sharp declines in biodiversity and an increase in agricultural land, greenhouse gas emissions, and usage of freshwater [15,16,17,18]. Beyond that, increasing incidence of non-communicable diseases (diabetes, cardiovascular diseases, cancer, chronic respiratory diseases and mental disorders) in the western world is alarming as they account for an estimated 86% of the deaths and 77% of the diseases [19].

Therefore, one of the world’s most urgent challenges is to produce and consume meat in a way that minimises environmental impact, i.e., to reach the UN Sustainable Development Goals (SDGs) [16,18], while ensuring that the growing world population can be fed in a healthy way. There is, meanwhile, an increasing agreement that the transition to reduced meat consumption towards a more plant-based nutrition, more precisely by consuming more vegetables, fruits, whole grains, legumes, nuts, unsaturated oil and only low amounts of (red and processed) meat [16,20], is a key feature in addressing major health and sustainability challenges. This is not a new finding, as it was already shown in the 1960s that, for instance, a Mediterranean diet, which is characterised by a high intake of plant-based foods, moderate intake of fish, and low intake of red and processed meat, can be useful in addressing both health and environmental issues [20]. In fact, plant-based diets are becoming more and more popular because of the numerous reported benefits. The adequacy and thus healthiness of meat-free diets for children was shown to be given if carefully planned [21,22,23,24,25,26].

Like in many other western countries, German children and adolescents consume too little plant-based food and far too many high-fat, animal-based foods such as meat and sausages: The majority of the children aged 6 to 11 exceed the recommendation in 2006 up to the double quantity—among boys at the age of 12 to 17 years 19% consume even more than the triple [27]. The nutritional situation of children and adolescents has improved in some aspects in recent years, but the overall picture of nutrition still shows a need for improvement: for meat and sausage products, a significantly lower consumption should still be aimed for [28].

Even with the awareness of what has to happen, it is not simple to implement an actual change in our eating behaviour. One of the most promising ways is the work with children—who learn to shape their eating habits in accordance with how they are stimulated—to step in and plant the seeds for a lifelong healthy and sustainable diet.

In response to calls to expand knowledge on the change towards a reduced meat consumption [18,29], and since food preferences and dietary habits are formed in childhood, this study contributes to the understanding of parent’s readiness to adopt a more plant-based diet for their children.

The aim of this study was to investigate the following research objectives: First, to examine whether the parent’s attachment to meat has an impact on their meal choice and, therefore, eating behaviour, including meat consumption, of their child. Second, to investigate the reasons associated with choosing a meaty dish for their child. Third, to explore and discuss opportunities and approaches to implement a meat-reduced diet.

## 2. Materials and Methods

A quantitative and partly qualitative data collection was carried out with the help of an online questionnaire to obtain data about the respondent’s attitudes, socio-demographic background, as well as details about the respondent’s child. A central part of the survey was the visual presentation of six classic dishes, each prepared in three different ways: meaty, reduced meat and no meat. For further details, please see the supplemented English translation of the survey. The questionnaire was developed using SurveyXact and accessible for three weeks in October 2020. The target group for this survey was parents living in Germany with children aged between 5 and 8 years. Prior to the main study, a pilot test with 8 participants was conducted in order to check for comprehensibility of the questions, missing answer options and duration of the whole questionnaire. Results and detailed feedback from the pilot study led to adjustments in terms of wording, i.e., rephrasing sentences and answer options, as well as additional answer options for vegetarians in questions asking for meat consumption behaviour (more precisely, including statements like “I do not consume meat” when asking for possible reasons for meat reduction, or “I do not purchase meat” when asking for purchasing behaviour of meat products). The 5-point Likert scales were changed to 7-point Likert scales according to the wishes of the participants, as they wanted more detailed gradation in agreement. One useful suggestion was to vary the order of photographs so that it is not always (A) meaty, (B) reduced meat, (C) no meat, which was implemented using permutation calculation.

### 2.1. Recruitment

Participants were recruited online via social media groups (for example Facebook, LinkedIn) and personal environment. Moreover, posters with a QR code linking to the survey were hung up at several places frequently visited by families in Nuremberg, Germany. Finally, 80 elementary schools throughout Germany (5 schools per federal state) were systematically contacted by telephone and e-mail and asked for cooperation in this research project. As an incentive to participate, each participant could get a sheet with informative links about children’s nutrition. Additionally, everyone could win one out of ten children’s cookbooks (“Kinderleichte Becherküche − 5 Messbecher + Rezeptbuch Band 6 − Gesund & Lecker”), financed by “Smag for Livet”, by providing their e-mail address.

The participants gave their consent to their answers being used for scientific purposes, and the obtained data was treated in accordance to the General Data Protection Regulation, GDPR (german *Datenschutz–Grundverordnung* [30]).

### 2.2. Questions

The questions were designed to explore the food choice of parents for their children. They covered, besides demographic background, topics such as food choice, the child’s diet (including meat consumption) and eating behaviour, as well as the parent’s diet and attachment to meat. The latter was investigated using the *Meat attachment questionnaire* (MAQ), which was developed and validated by Graça and colleagues in 2015 in order to expand the knowledge in the area of consumer’s willingness to shift from a meaty diet towards a more plant-based diet [31]. It assesses the positive bond of a person towards meat consumption and until now, it was used for studying reductions in meat consumption and the adoption of plant-based diets [31,32,33], as well as for a study on alternative proteins [34]. Moreover, parts of the *children’s eating behaviour questionnaire* (CEBQ), developed by Wardle and colleagues in 2001 to assess the eating behaviour of a child using statements and a five-point Likert scale ranging from 1 = *never* to 5 = *always* [35], were utilised. The statements evaluating *Food fussiness*, *Enjoyment of food*, and *Satiety responsiveness* were applied because it was previously shown that these three scales predict children’s food preferences [13]: *Food fussiness* statements (for example: *“My child refuses new foods at first”*) show if a child is very selective about new foods, which can therefore be an indicator for food neophobia. *Enjoyment of food* (for example: *“My child looks forward to mealtimes”*) reflects the general interest in eating. In previous studies, it was shown that there are positive associations between the *Enjoyment of food* scale and fruit and vegetable intake [36] and meat intake [13]. *Satiety responsiveness* (for example: *“My child leaves food on their plate at the end of a meal”*) gives information about a child’s ability to react to satiety signals, e.g., a reduced food intake to compensate for a prior snack [35]. With a low responsiveness, the energy intake will not be regulated, which leads to a higher risk of overeating and eventually to obesity [37,38].

### 2.3. Picture Stimuli Instead of Real Food

Using a hypothetical simple meal choice, it was asked as a which out of three meals (presented as photographs with a neutral description) the participant prefers to prepare for their child with a follow-up question (CATA) about the respective reasons. An alternative to real food—which can be expensive and time-consuming to prepare—is using pictures of food instead, and in fact, applied in studies with children, pictures were revealed to have no statistical significant difference in reliability compared to real food [39]. Picture stimuli have been used in many studies to investigate preferences or willingness to buy food [12,40,41,42]; some studies used both pictures and real food [43,44]. Hence, photographs were used in the present survey instead of a description of the dish solely.

### 2.4. Dishes

**Figure 1:**
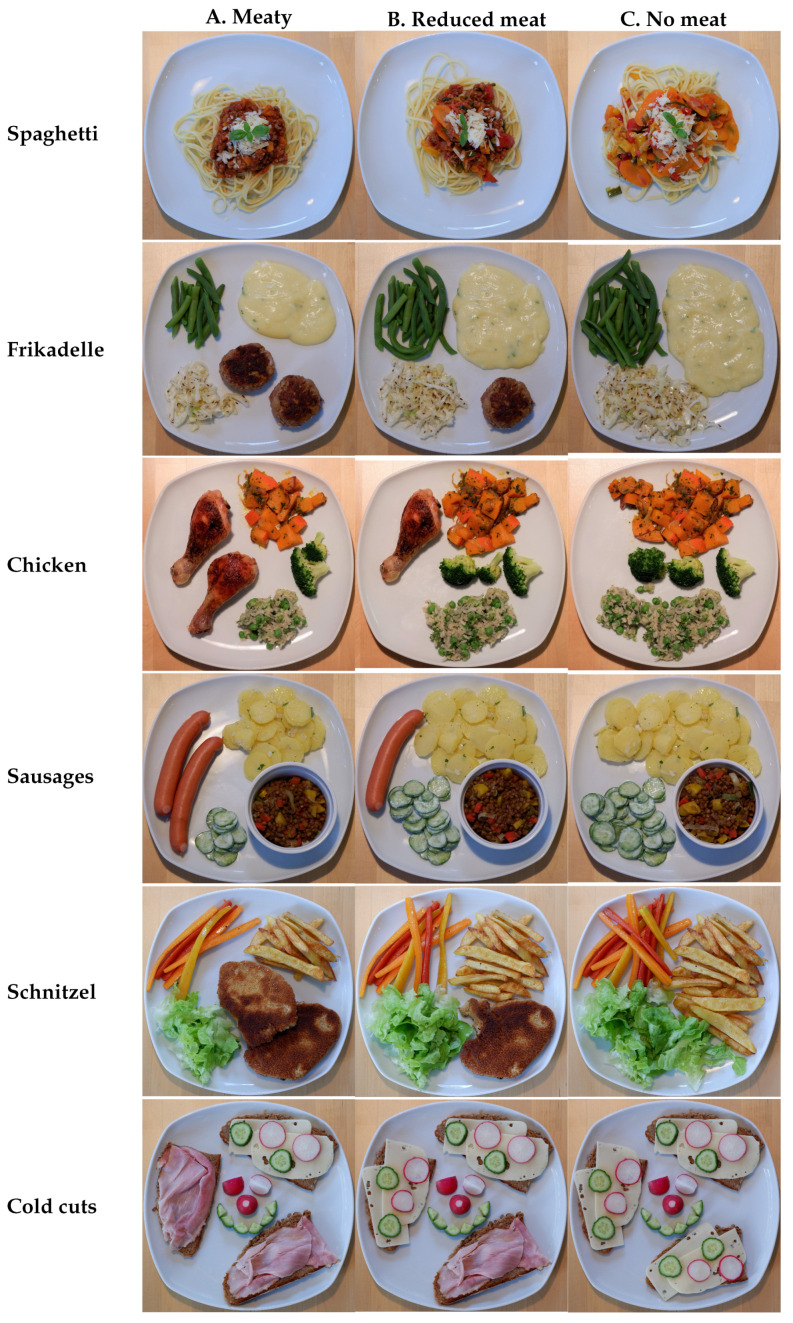
Photographs of the 6 dishes, left to right: (**A**) meaty, (**B**) reduced meat, (**C**) no meat.

The focus was further on well-known, traditional meals with meat. Based on personal interviews with parents of children aged 5–8 years prior to the development of the questionnaire, combined with recommended recipes for children by the DGE [45], a selection of the following six dishes was created, please see Figure 1: Spaghetti Bolognese, Frikadelle, chicken drumsticks, sausages, Schnitzel, and cold cuts. The six dishes were chosen in three variations, respectively: One variant with meat (A), one with reduced meat (B), and one no-meat variant (C). Attention was paid to the following three points: First, the ingredients only changed from A to C in quantity, no new ones were added. More precisely, the meat amount was first reduced by the half and then omitted completely. Second, by reducing the meat by half in variant B, the vegetable content was increased by half. In variant C, the vegetable content was increased by the same amount as from A to B. Third, so that the no-meat variant C still consists of three components, three vegetable items were also used in variant A. The exact recipes are available from the authors upon request.

The dishes were served on white square plates with the dimensions 26 × 26 cm (diameter 28 cm) and photographed with the same background (wooden table) at about the same time of day to ensure similar lighting conditions. The used camera was Fujifilm X-T100 with a camera lens of 50–230 mm. The order of the meals was fixed for all participants. The three options were fixed as well, however, in prior mixed up order using permutation (Spaghetti: A–B–C; Frikadelle: A–C–B; chicken drumsticks: B–A–C; sausages: B–C–A; Schnitzel C–A–B; cold cuts C–B–A).

### 2.5. Data Analysis

The analysis of the data was conducted using RStudio (Version 1.1.463, © 2009–2018 RStudio, Inc. Boston, MA, USA). To determine the significance of the results, an α-level of 0.05 was applied. Descriptive statistic was used to analyse the respondent’s demographic background. Socio-demographics are presented as proportion, and scores for Likert scales are presented as means with standard deviation. Body mass index (BMI) was calculated based on data for body weight and height. Individuals were classified in underweight (BMI ≤19), normal weight, and overweight (BMI ≥25) [46].

Cronbach’s α was computed for CEBQ and MAQ to assess internal consistency. Following Wardle and colleagues [35], the applied CEBQ items were grouped into the subscales *Enjoyment of food, Food fussiness* and *Satiety responsiveness*, and, respectively, summed up with respect to the reverse counted items. The mean for each respondent’s child was calculated. By calculating the mean scores for the MAQ dimensions (*Hedonism, Affinity, Entitlement*, and *Dependence*) with respect to the reverse scored items, the respondent’s attachment to meat was evaluated. A low score represented low meat attachment (MA) and a high score high MA.

In order to investigate the study population in more detail, the study population was divided according to their MA scoring. Circus and colleagues applied the Ward’s cluster analysis in their study about alternative proteins from 2018 to identify two clusters, namely low and high MA [34]. However, a cluster analysis was not practicable in this study, due, among other reasons, to the resulting insufficient number of participants per cluster. Hence, it was assumed that the majority of the population has an intermediate attachment to meat and that people with a low or particularly high attachment are the exception. Since there is no recommendation for a possible grouping, the interquartile range (IQR) was used for a precise and systematic classification. Hence, the scoring in the MAQ was utilised to divide the study population into three groups according to the IQR: low MA (25%), medium MA (50%) and high MA (25%). Characterisation of the three groups was carried out with ANOVA and Pearson’s $\chi^{2}$-tests to investigate associations between the level of MA (as independent variable) and the decision for a dish, attitudes, knowledge, social barriers and demographic background (respectively, as dependent variable). For a $\chi^{2}$-test, the expected frequencies in each cell must be greater than 5, otherwise the results of the test will be somewhat inaccurate. If this condition was not met, a $\chi^{2}$-test with simulated *p*-value (based on 2000 replicates) was used instead. Bonferroni post hoc tests for $\chi^{2}$ (with simulated *p*-value) have been applied to significant outcomes to assess the given differences. In addition, for significant ANOVA outcomes, a TukeyHSD post hoc test was applied. A pairwise independence test compared the variables of interest.

Moreover, a linear regression analysis was carried out using the function lm() (standard installation in RStudio, no additional package required) to investigate how 1. the age of the child affects the child’s eating behaviour, and 2. the parent’s MA affects the child’s eating behaviour. To answer the question of how the parent’s MA affects the parent’s hypothetical choice of a dish for the child, ANOVA and TukeyHSD post hoc test was applied.

## 3. Results

Of the 107 individuals participating in the study, 91 finished the online survey, and 16 cancelled before finishing it. One respondent was further excluded, as he or she only wanted to “click through the survey”. This resulted in 90 valid responses for the data analysis. In the following, a description of the study population in terms of their attachment to meat will be provided, as well as results to their meat consumption. Furthermore, the results on meat substitutes are presented, followed by the description of the child’s nutrition. The outcomes on the preferred option of a dish are described next. Lastly, the findings for possible reasons for meat reduction are demonstrated.

### 3.1. Grouping According to Attachment to Meat

In order to investigate the study population (n = 90) in more detail, the respondents were grouped according to their attachment to meat similar to Circus and colleagues in their study about alternative proteins from 2018, who applied the Ward’s cluster analysis to identify two clusters, namely low and high attachment to meat [34]. However, in this analysis, the grouping was based on the IQR (13 to 18.36) of the study population’s MA scoring (median: 16.32): low level of MA was reached with a score <13.00, while high MA was defined having a score >18.36. Hence, 22 participants were identified as having low MA, 45 as having a medium MA and 23 as having high MA, please see Table 1.

### 3.2. Description of the Study Population

An overview of the study population’s description is visible in Table 1. The participants were primarily mothers (n = 77, 85.6%). The pairwise test of independence revealed that in the high MA group the proportion of fathers was significantly higher than that of mothers (p=0.01). Almost all participants (n = 63, 70.0%) had one child aged 5–8 years. The age of respondents was 37 ±5.2 years with a significant age difference of +3 years on average in the high MA group compared to the medium MA group (p=0.045). The age of the child to whom the survey referred was about evenly distributed, as there were n = 25 5-year-olds, n = 20 6-year-olds, n = 26 7-year-olds and n = 19 8-year-olds. More than half of the participants (n = 50, 55.6%) were living in a city, n = 26 (28.9%) in suburban area and n = 14 (15.6%) on the countryside. The educational level was predominantly high (university degree) (n = 53, 58.9%). Post hoc analysis showed that the low MA group included significantly more people with higher education (p=0.012), while the high MA group included significantly fewer people with high education (p=0.026) and more people with moderate education (p=0.015). Most of the respondents were workers (n = 73, 81.1%). The respondent’s average BMI was 23.9 ± 3.4 with no significant difference between males and females, which is considered as a healthy body weight. Most of the participants defined themselves as omnivore (n = 54, 60%) or flexitarian (n = 28, 31.3%), while n = 3 (3.3%) described their eating habits as vegetarian and vegan, respectively, and n = 2 (2.2%) as pescetarian. As expected, the dietary identity reflected the level of MA significantly (p<0.001): members of the high MA group categorise themselves as omnivores (p<0.001) and less as flexitarians (p=0.007), and low MA members mainly as flexitarians (p=0.03) and less as omnivore (p<0.001). No significant differences between the three MA groups were found for living area, occupational status, BMI or number of children aged 5–8 years.

### 3.3. Attachment to Meat

The internal reliability (provided with Cronbach’s α) for all subscales and the global scales of the MAQ was given: *Hedonism*: 0.90; *Affinity*: 0.90; *Entitlement*: 0.88; *Dependence*: 0.91, *Global scale*: 0.95 [47]. The mean scores for the MAQ subscales, as well as the sum of those, were calculated to evaluate the individual’s level of MA. The subscales showed a normal distribution for *Hedonism* (mean 3.56 ± 1.52, median 3.5, IQR 2.5–4.25) and *Dependence* (mean 3.75 ± 0.54, median 3.8, IQR 3.4–4), a left skewed distribution for *Affinity* (more agreement than disagreement with the statements, mean 5.01 ± 1.55, median 5.25, IQR 4–6.25) and a slightly right skewed distribution for *Entitlement* (more disagreement than agreement with the statements; mean 3.47 ± 1.65, median 3.33, IQR 2.08–4.67). The reached level of the *Global scale* was 15.80 ± 4.25 with a lowest mean of 6.90 and a highest mean of 25.35 and was normally distributed.

### 3.4. Meat Consumption: Amounts, Frequencies and Purchasing Behaviour

To gain insights about the respondent’s meat consumption, the frequency and quantity of meat consumption was asked for. For the latter, the respondent had to enter the consumed grams per week in an open box to avoid potential influence from using fixed response categories. The results are explained in the following.

#### 3.4.1. Amounts of Meat Consumed

When comparing the consumed amounts with the dietary recommendations of 300 to 600 g of meat per week (by DGE), 38 participants were found to consume less than the lower limit of 300 g while 21 consume more than the upper limit of 600 g per week. The study population’s indicated quantities vary considerably and range from 0 to 3000 g. However, such high amounts as 3000 g are only eaten by the minority—half of the study population eats between 157.5 and 600 g per week (median: 250 g, mean: 479.4 g), which is within a moderate range. It was investigated whether there is a difference in meat consumption of men and women, however, a t-test revealed no significant differences (p=0.160).

The average meat consumption of the respondent’s child was 363.9 g per week, however, values between 0 and 3000 g were entered again. For German children, the recommendations are approximately 35 g per day for children aged 4 to 6 years, and 40 g for children aged 7 to 9 [27]. This results in an approximate recommendation of 260 g per week for the children of interest in this study. Hence, the median of the children’s consumed meat amounts was 250 g (IQR 150–500), which is almost exactly the official recommendation.

#### 3.4.2. Frequency of the Parent’s and Child’s Meat Consumption

The frequency of meat consumption is shown in Table 2 and was mainly 2–3 times a week and 2–4 times a month (respondent: n = 58, 64.4%; child: n = 56, 62.2%), while n = 21 (23.3%) respondents and n = 30 (33.3%) children consume meat or meat products daily, and n = 11 (12.2%) respondents and n = 4 (4.4%) children never. It was further investigated if the respondent’s level of MA was associated with frequency of meat consumption. As expected, significantly less respondents with low MA consume daily meat (p<0.001), they rather consume it once a month (p<0.001) or never (p<0.001). Respondents with medium MA consume it more frequently (2–3 times a week) (p=0.031) and with high MA in particular daily (p<0.001).

In terms of meat consumption frequency of the child, significantly less children of parents with low MA consume daily meat (p=0.024), they consume it rather 3–4 times a month (p=0.003) or never (p=0.012). Notably was that children of parents with low MA consumed more frequently meat or meat products (n = 3 for daily, n = 2 for 2–3 times a week) than their parent did (n = 0 for daily, n = 6 for 2–3 times a week).

### 3.5. Purchasing Behaviour of Organic Versus Conventional Food

Furthermore, the purchasing behaviour (organic versus conventional) with regard to meat and plant-based food was asked, significant differences were found and the results are shown in Table 3. Compared to the respective other groups, a significant higher number of individuals (n = 8) with low MA purchased no meat (p<0.001), while a significant larger number of individuals (n = 5) of the high MA group purchased conventional meat (p=0.012). Similar results were found for plant-based food, with significant difference between low and high MA (p=0.004) as individuals with high MA purchased significantly more conventional (p=0.008) and significantly less organic (p=0.112) products.

### 3.6. Meat Substitutes as an Alternative

**Figure 2:**
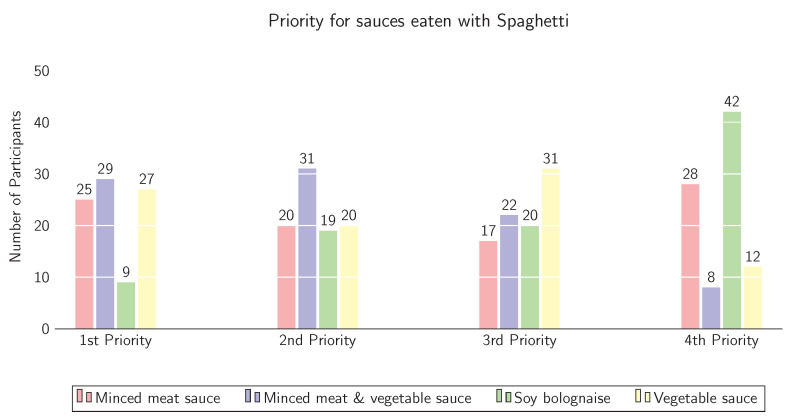
Arranged preferences for spaghetti with minced meat sauce, minced meat & vegetable sauce, soy bolognaise and vegetable sauce, 1 = favourite, 4 = least favourite.

For frequency of meat substitute consumption, there was no difference between the three levels of MA as almost all participants never consume meat substitutes. This consumer behaviour is mirrored in the result to the question *“Please arrange the following four dishes (Spaghetti with minced meat sauce, minced meat & vegetable sauce, soy bolognaise and vegetable sauce) according to your own preference for consumption. (1 = favourite, 4 = least favourite)”*, visualised in Figure 2, where soy bolognaise (green bar) was chosen by 42 participants as the 4th priority and by 20 participants as the 3rd priority. Minced meat & vegetable sauce was voted as 1st and 2nd priority (n = 60) over the other sauces.

### 3.7. The Child’s Nutrition

Information about the children’s diets were investigated using several approaches. First, the respondent had to prioritise four characteristics of a meal for their child according to importance. Then, five single statements were provided, where the parent had to agree with a 7-point Likert scale. Here, it was investigated if the agreement varied according to the MA grouping. Moreover, the CEBQ gave details about the child’s eating behaviour in terms of enjoyment and fussiness when eating, as well as response to satiety. The impact of the child’s age and the parent’s MA was investigated by linear regression analysis.

#### 3.7.1. Important Characteristics of a Meal for the Child

**Figure 3:**
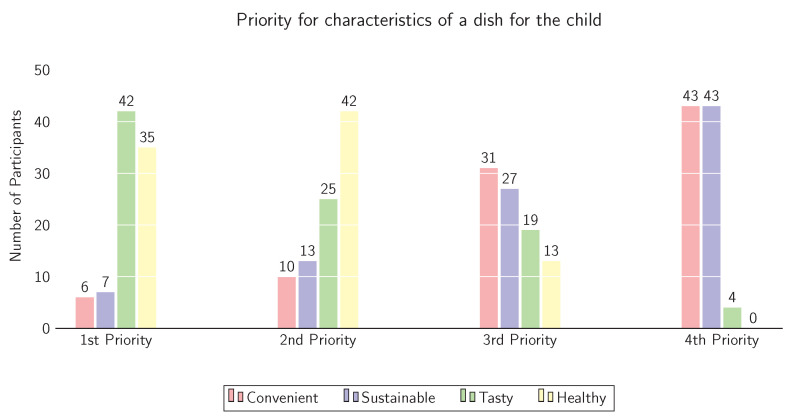
Arranged importance of four characteristics of meals for the respondent’s child, 1 = most important, 4 = less important.

The respondent was asked to rank the following four characteristics of meals for their child in order of importance (1 = most important, 4 = less important): *tasty*, *sustainable*, *easy to prepare*, and *healthy*. The result is visualised in Figure 3 with *tasty* (n = 42) being the first priority, closely followed by *healthy* (n = 35). *Convenience* and *sustainability* was found to be less important to the parent when deciding for a dish, since almost all participants rated either one or the other as 4th priority (n = 43, respectively).

#### 3.7.2. Single Statements to Agree to

With the help of a 7-point Likert scale, it was asked how much the participant agrees (1) or disagrees (7) to five single statements: perceived healthiness of the child’s diet (representing *knowledge*), also in regards to high meat consumption, evaluation of the nutritional relevance of meat in the child’s diet (*dependence*), willingness to reduce the child’s meat consumption (*willingness*) and perceived social support when doing or planning so (*social barriers/facilitators*). There were no differences between the three levels of MA for *knowledge*, since the majority felt the nutrition of their child as healthy (mean: 2.48 ± 1) and did not agree that the child should eat less meat (mean: 5.02 ± 1.5). The same was true for *willingness*, since everyone could imagine reducing the child’s meat consumption in future (mean: 3.29 ± 1.6), and for *social barriers/facilitators*, as the majority claimed to be rather supported from their social environment when giving the child no meat any more (mean: 4.31 ± 1.93). The only difference was found for the statement covering *dependence* (mean: 3.88 ± 1.73), where respondents with low MA think that meat is less important for a child’s healthy development (p<0.001).

#### 3.7.3. Children’s Eating Behaviour Questionnaire

Information about the child’s eating habits was obtained through the CEBQ. The three applied dimensions of the CEBQ were normally distributed and reached good levels of internal reliability with Cronbach’s α of 0.87 for *Enjoyment of food*, 0.92 for *Food fussiness*, and 0.76 for *Satiety responsiveness* [47]. The means with respective standard deviations were 3.83 ± 0.79 for *Enjoyment of food*, 2.88 ± 0.87 for *Food fussiness*, and 2.88 ± 0.7 for *Satiety responsiveness*, each on a 5-point Likert scale ranging from *Never = 1* to *Always = 5*.

#### 3.7.4. Impact on the Child’s Eating Behaviour

By means of linear regression, it was examined in what way the child’s eating behaviour is affected by 1. the age of the child and 2. an increase in the parent’s MA. No significant results were found for a possible effect of the child’s age on all scales: *Enjoyment of food* (β=0.09±0.30, $R^{2}=-0.01$, Df=88, p=0.757), *Food fussiness* (β=−0.12±0.49, $R^{2}=0.01$, Df=88, p=0.803) or on *Satiety responsiveness* (β=−0.29±0.31, $R^{2}=0.00$, Df=88, p=0.356). In addition, no effect of the parent’s MA could be revealed on *Enjoyment of food* (β=0.05±0.07, $R^{2}=-0.01$, Df=88, p=0.489) or *Food fussiness* (β=−0.22±0.13, $R^{2}=0.21$, Df=88, p=0.090). Only for the child’s *Satiety responsiveness* significant results were found as with increased attachment to meat the level of *Satiety responsiveness* decreased significantly (β=−0.27±0.13, $R^{2}=0.04$, Df=88, p=0.044). Thus, the children of parents with higher attachment to meat show signs of satiety more slowly. R-squared revealed that 4% of the variance in the obtained results for *Satiety responsiveness* can be explained by the parental scoring in the MAQ.

### 3.8. Preference for a Dish

**Figure 4:**
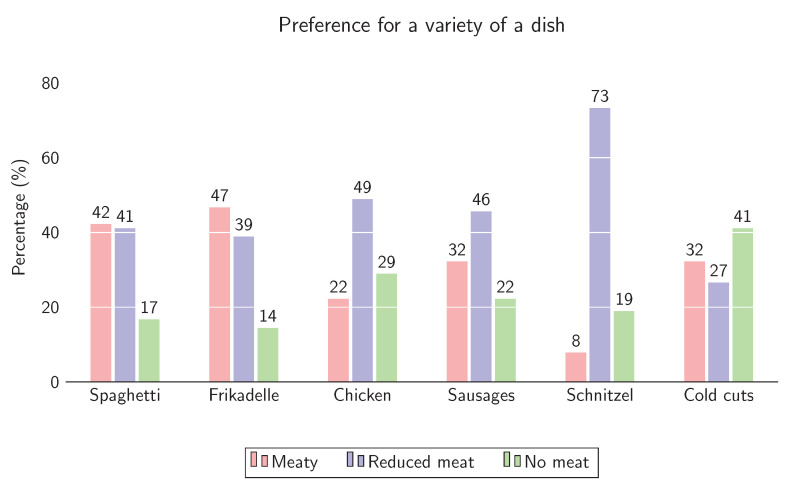
Proportions (in %) of participants choosing the preferred variety (meaty, reduced meat, no meat) of a dish (Spaghetti, Frikadelle, Chicken drumsticks, sausages, Schnitzel, cold cuts).

For each dish, the parent had to choose the preferred variety for their child, where the results are illustrated in Figure 4. For Spaghetti, there must be meat as an ingredient in the sauce for most of the respondents (42.2% chose *meaty* and 41.1% chose *reduced meat*). Only 16.7% preferred the pure vegetable sauce for their child. For the second dish, two Frikadelle were preferred by narrow majority (46.7%) over one (38.9%). Again, the *no meat* variety containing only mashed potatoes, green beans and cole slaw was the least preferred (14.4%). The *reduced* version of chicken drumsticks with one drumstick was preferred by 48.9%, followed by the *no meat* variety with 28.9% and the *meaty* variety containing two drumsticks (22.2%). For sausages, the majority of the parents chose the *reduced* variety of one sausage (45.6%), followed by two sausages (32.2%) and the *no meat* option. In general, the most chosen option of all varieties was the *reduced* version of Schnitzel (73.3%) and the least chosen variety was the *meaty* version of Schnitzel (7.8%), while 19.9% chose the *no meat* variety. With the last dish, slices of bread with cold cuts, 41.1% preferred the *no meat* version, 32.2% the *meaty* version, and 26.6% the *reduced meat* version.

#### 3.8.1. Parent’s Attachment to Meat Affects Their Hypothetical Decision for a Dish

By dividing the study population according to their scoring in the MAQ, the hypothesis was tested whether belonging to a group of low, medium or high MA was associated with the choice of a dish’s variety. The applied null hypothesis ($H_{0}$) was that there is no association, with an alternative hypothesis ($H_{A}$) that the level of MA is associated with the preference for a dish. Significant results were obtained for all dishes (Spaghetti: p=0.007, Frikadelle: p=0.008, chicken drumsticks: p=0.012, sausages: p=0.029, Schnitzel: p=0.001, cold cuts: p<0.001), hence the hypothesis can be accepted in all cases.

To address the question to which extend the parent’s attachment to meat affects the meal choice for their child the grouping of parents according to MA was deferred, and attachment to meat was used as a continuous variable to study the general effect on their hypothetical meal choice.

- The difference between **reduced meat** and **meaty**: For all dishes, the level of MA was overall 1.6 points lower (p<0.01) when deciding for a reduced meat option compared to the meaty option. However, the only significant change in MA scoring in terms of the single dishes was found for cold cuts with a change of −3.0 units (p≤0.05) when choosing the reduced meat version instead of the meaty dish.
- The difference between **no meat** and **meaty**: For all dishes, the level of MA was 4.4 units lower (p<0.001) when deciding for a no meat option compared to the meaty option. In terms of the single dishes, significant results were obtained for each dish (Spaghetti: −5.3, p<0.001; Frikadelle: −4.4, p<0.01; Chicken drumsticks −4.7, p<0.01; Sausages −3.5, p≤0.05; Schnitzel −7.1, p<0.01; Cold cuts −4.7, p<0.001).
- The difference between **no meat** and **reduced meat**: For all dishes, the level of MA was 2.8 units lower (p<0.001) when deciding for a no meat option compared to the reduced meat option. However, only for Spaghetti (−4.1, p<0.001), Frikadelle (−3.4, p≤0.05) and Schnitzel (−4.3, p<0.001) were significant results obtained.

#### 3.8.2. Stated Reasons behind the Parent’s Choice of a Dish

**Figure 5:**
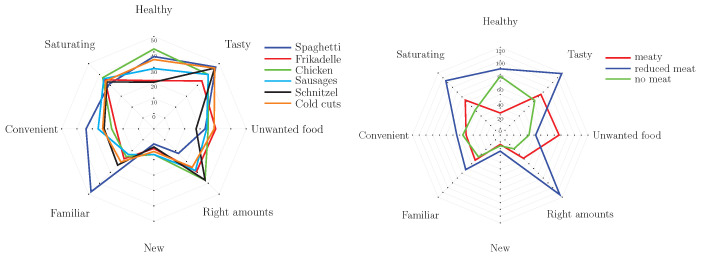
Radar diagram showing the total counts of reasons for choosing a variety (meaty, reduced meat, no meat) of the respective dishes (Spaghetti, Frikadelle, chicken drumsticks, sausages, Schnitzel, and cold cuts). Left: Reasons in term of the dish; right: Reasons in term of the variety.

Great interest was paid to the reasons for the respondent’s choice. A total of 8 reasons were given, of which the participant was asked to check all that apply (CATA), please see Figure 5 for a graphical overview of the outcome. In addition, there was the possibility to give other reasons that were not listed by using an open box answer. The overall most often chosen reasons were *tasty* (259 times, with n = 35–48), *saturating* (212 times, with n = 32–38) and *healthy* (190 times, with n = 21–43). On the whole, the reasons did not distinguish strongly in the comparison of the individual dishes, please see Figure 5 to the left. However, comparing the three options (meaty, reduced meat and no meat), it was observed that *healthy* was a frequently chosen reason for the no meat option, please see Figure 5 to the right. *Right amounts* was a popular reason for the reduced meat option. Since the reduced meat option was the most chosen option overall, a balanced serving style, i.e., right amounts of all food items, is of higher relevance to many parents. For the meaty option, *least amount of what my child does not eat* was chosen most frequently. The vegetable proportions on the meaty dish are the smallest compared to the other options, which indicates that the respective child does not like one or more of the vegetables on the plate. Hence, it was not solely the meat amount that was the tipping point, it was also the vegetable that affected the parental food choice here.

### 3.9. Possible Reasons for Meat Reduction

**Figure 6:**
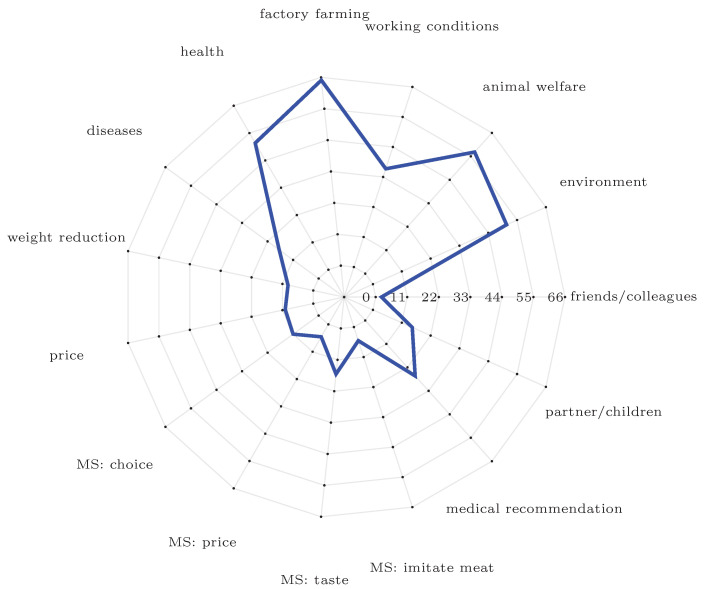
Radar diagram showing counts for chosen possible reasons for meat reduction. (Note: MS = Meat substitutes. *MS: choice* = More choice of MS, *MS: price* = cheaper MS, *MS: taste* = tastier MS, *MS: imitate meat* = MS which imitate meat better.)

A total of 15 reasons were presented, all of which the participant was asked to check all that apply (CATA), please see visualisation in Figure 6. In addition, there was the possibility to give other reasons that were not listed by using an open box answer. Moreover, there were two further options, one in case the participant cannot imagine reducing their meat consumption and one for the case that the participant does not consume meat. The latter was selected by five participants. That a reduction in meat consumption is unimaginable was only clicked once. Four of the given reasons were covering meat substitutes (MS): *tastier* meat substitutes (n = 17), followed by *more choice* of meat substitutes (n = 12), *cheaper* meat substitutes (n = 6), and meat substitutes that *better imitate meat* (n = 6). One participant provided a fifth aspect by choosing "others" and writing *"Meat substitutes with fewer additives"*, and thus emphasised the (questionable) *health aspect* of meat replacement products. Of all possible reasons, those concerning optimised meat substitutes were clicked relatively rarely while reasons related to meat production were clicked often. The most frequent reason was *factory farming* (n = 66), the second most frequent reason was *animal welfare* (n = 58), followed by *environment* (n = 52) and *health* (n = 52), *working conditions* (n = 37) and *medical recommendation* (n = 27). Reasons such as *diseases transmitted by animals* (n = 18), wishes of the *partner and children* (n = 16), *increased price of meat products* (n = 11), to *reduce body weight* (n = 10), or the fact that all *friends/colleagues reduce or avoid meat* (n = 3) were clicked less often.

### 3.10. Open End Comment Boxes

Parents were asked at the end of the survey to write down their thoughts and comments about the topics “children’s nutrition” and “meat reduction”, which was done by 21 participants. These comments were analysed and based on their content grouped into four areas that parents found important to comment: First, parents explaining difficulties, e.g., having troubles implementing a healthy diet for their children. Second, describing the nutritional importance of meat for a child’s balanced diet. Third, about the high importance of meat’s organic origin. Fourth, about the child’s preferences and aversions of certain food.

## 4. Discussion

### 4.1. The Parental Attachment to Meat

The examination of parent’s attachment to meat using the MAQ resulted in a normal distribution of MA. This made it possible to divide the study population into three groups for further investigation. One option of dividing would have been to split the population by a third to obtain groups with similar amount of members. However, it was considered more plausible that the general population had a “normal relationship” to meat and that people with a particularly high or low propensity were more likely to be in the minority. As the three groups distinguished themselves significantly in many other aspects surveyed, it can be assumed that the applied grouping according to interquartile range was reasonable.

The obtained data for demographic background revealed that the majority were mothers, which was consistent with prior studies, where the study population consisted of more women than men [31,33,48]. Although there were only 13 fathers, a significant difference was found in terms of MA: Men were found to score higher. Graça and colleagues have already shown this result in their studies in 2015 and 2016 [31,33]. In addition, it was prior shown that more women follow a vegetarian diet than men do [48,49,50], and therefore this result was as expected. In terms of dietary identity, in 2016, meanwhile, 4.3% of the German population (6.1% of women and 2.5% of men) aged 18–79 followed a vegetarian diet [49]. The proportion of vegans or vegetarians is rising, as in 2013 there were only 3.7% vegans or vegetarians [51], which was already a doubling of the numbers from 2006 [52]. In fact, these proportions are again consistent with the obtained data in the survey showing that 6.7% followed a plant-based diet. Having in mind that the majority of this study population is female and has a university degree, a slightly higher proportion of vegetarians and vegans in this survey compared to the general German population can be explained as females [31,49,50] and individuals with a higher education are more likely to follow a plant-based diet [49,50,53]. Here, it should be emphasised that in the current study, a low MA was going along with high education (p=0.026) and, on the contrary, high MA with moderate education (p=0.015).

The purchasing behaviour of meat and plant-based food was found to distinguish amongst the three groups as high MA was associated with purchasing conventionally rather than organically produced food. It was shown before that low consumption of meat (and high consumption of fruit and vegetables) was associated with high stated purchase of organic food items [54]. Furthermore, the dietary identity of parents also had a similar effect on purchasing behaviour of organic/conventional food products, as it was shown in the VeChi Study from Weder and colleagues (2019) that more than half of the examined vegan parents (51.4%) purchase organic food products, followed by vegetarian parents (36%) and omnivore parents (12.3%) [55].

### 4.2. Meat Consumption Lower Than Expected

The DGE recommends a meat consumption of 300 to 600 g per week for adults [56], which is similar to other recommendations such as from the World Cancer Research Fund International [57], and approximately 260 g for children aged 4 to 9 years [27]. Although the amounts reported for meat consumption ranged from 0 to 3000 g, it could be seen that the average amount of meat consumed by the study participants and their children was around 300 and 250 g, respectively, per week. The results are lower than expected, as prior official data collection indicated a meat consumption of the German population higher than the recommendations: men consume 103 g and women 53 g per day [52,58,59]. Therefore, it was expected that men consumed approximately 720 g per week and women 371 g. In fact, the average meat consumption of men was 753 g (median: 500 g) and for women 433 g (median: 300 g), which would be consistent with the above references, but this obtained result was not significant (p=0.162). However, this could be due to the fact that the study population included only 13 men versus 77 women. Interestingly, the entered values of consumed meat amounts per week differed markedly, as one mother reported a consumption of 3000 g per week for herself and 3000 g for her 5-year old child. This could be due to overestimating the actual consumed amounts, which is a known interference factor in food frequency surveys [60]. However, the same paper found out that processed meat was the food item most underestimated by consumers [60]. In order to prevent incorrect estimates of the weekly consumed amounts of meat, examples for the weight of meat items were provided to the parents. Nevertheless, the applied assessment is still an imprecise measurement and only an estimation of past food intake [46], hence, it must be noted here that this data collection of meat consumption was not the optimal, but a practical one for an online survey. The median of consumed meat amounts by children with 250 g per week are almost exactly according to the German recommendation. However, looking at the broad IQR of 150 to 500 g, then one can conclude that too much meat is consumed by German children, as 500 g is almost twice the recommendation.

As a second indicator of meat consumption, the frequency of meat intake was asked in addition to gain more information on meat consumption. Even though the majority consumes meat and meat products regularly (2–3 times a week and 2–4 times a month), about a quarter of the population consume it daily. Interestingly, there are more children consuming meat products daily (n = 30, 33.3%) than parents (n = 21, 23.3%). The underlying reasons can only be guessed, as on the one hand it could be due to the fact that children in Germany also eat lunch outside their homes, for example, day care centres cook for lunch or provide a hot meal via catering. Primary schools also offer the possibility of eating a warm meal at lunchtime. Up to 5 days a week, the child could therefore consume meat for lunch without the parents being able to estimate the amount. On the other hand, it could be due to the fact that it is still a widespread opinion that children need meat for their healthy development, as confirmed by the significant high agreement to the statement “*Meat and meat products are important for my child’s development*” (mean 3.88 ± 1.73 on a 7-point Likert scale where 1 = Totally disagree and 7 = Totally agree, p<0.001) and in addition by a Slovenian study from 2013, where respondents stated that a vegetarian diet brings health benefits for adults, but is not appropriate for children [61]. However, this perception is now outdated, as a well-planned meat-free diet for children can be healthy [21,22,23,24,25,26,62] and even beneficial for a healthy diet in adulthood, as healthy eating habits are formed in childhood.

### 4.3. Children’s Eating Behaviour and Aspects Influencing It

The results for the children eating behaviour showed normal distribution of the subscales with low standard deviations, indicating that the children had comparable eating behaviour, which therefore could not be utilised to differentiate the children. The linear regression analysis revealed that there are no significant effects of the child’s age on the three subscales. Prior studies showed that age is a predictor for preferences [13] and was therefore expected to be associated with child’s eating behaviour. In fact, it was seen that the scale *Satiety responsiveness* and *Enjoyment of food* decreases with age [63], and *Food fussiness* increased with the child’s age (range: 1–6 years) [63]. However, Wardle and colleagues found an increase of *Enjoyment of food* and *Food responsiveness* with increasing age (range: 3–8 years) [35]. Thus, it is of interest that in this study age (range: 5–8 years) had no effect on the three applied dimensions of the CEBQ.

The parent’s MA had almost no effect on the child’s eating behaviour, only a significant effect on *Satiety responsiveness* as with increased MA the child’s responsiveness decreased significantly (β=−0.27±0.13, p=0.044). Since high levels of MA was associated with high consumption of meat, it can be concluded that with increased meat consumption by the parents, the child is slower to show signs of satiety. In addition, the obtained R-squared revealed that 4% of the total variance in *Satiety responsiveness* can be explained by MA scoring ($R^{2}=0.04$). This might first sound not much, however considering the multitude of factors influencing a child’s eating behaviour, 4% is a share that should not be neglected. It was shown that if a child has a decreased responsiveness to satiety, it is more likely to be overweight [35]. The same was shown to be true for an increased meat consumption as this was associated with obesity [64]. Since eating habits are formed in childhood, a correlation here might be that high meat consumption in the respondent’s childhood was carried over to adulthood.

An increased MA of the parents was further associated in this study with increased meat intake of the child, which elsewhere was shown to be accompanied by reduced consumption of vegetables [27,49,53]. This result is therefore consistent with the results of Russell and colleagues (2016), where *Satiety responsiveness* was associated with a reduced liking for vegetables [13] and therefore less consumption of vegetables. Another indicator for pointing to the link between reduced vegetable consumption and increased meat consumption was the following: respondents were asked to give reasons for their decision of a dish and the provided answer “*The chosen dish has the least amount of what my child does not eat*” was used to find out if they did not like any of the food items on their plate and therefore made the choice based on this thought. In fact, this reason was most likely to be clicked if the meaty dish was chosen, implying that the respective child does not like one or more of the vegetables on the plate.

Another insight about the child’s nutrition quality was provided by the importance of a meal’s characteristic, i.e., parents stated that *tastefulness* of their child’s meal is the most important consideration, closely followed by *health*, while *sustainability* and *easy preparation* are less important. This result is consistent with the outcome of Murimi and colleagues study with adolescents from 2015, where taste, food appearance and familiarity were the main influences for food choice [65]. This indicates that parents should be more opportunities provided to learn about healthiness of a plant-based diet in particular for children. Cooking skills and ideas for tasty recipes should also be communicated in order to increase the selection of possible everyday meals. It was shown that an increase in parents’ cooking skills confidence was associated with a decrease in the child’s consumption of ultra-processed foods [66] and had therefore an impact on the healthiness of the child’s diet. In general, having cooking skills correlates positively with vegetable consumption [67] and thus this facet should be included if one wants to shape children’s nutrition in a more sustainable and healthier way. Especially public education and social marketing campaigns could provide information and recipes in different formats to appeal not only parents but other consumer segments to increase acceptability of plant-based diets [68].

### 4.4. Hypothetical Preference for a Dish Option

In addition, the parent had to choose out of three possibilities (meaty, reduced meat, no meat) the preferred option of a dish for their child. The parent’s MA was significantly associated with the choice of a dish variant: high attachment lead to choosing a more meaty dish. This result was consistent for all six dishes and as expected, since high attachment to meat was associated with high preferences for meat [31].

When comparing the three options (meaty, reduced meat and no meat) in terms of MA, the difference between choosing the meaty option and the no meat option was for all dishes highly significant, as increased MA was clearly associated with preference for a meaty dish. However, comparing the reduced meat option with the meaty option, the difference was not consistently demonstrable, implicating that although the participant wants meat in the dish as an ingredient, the quantity is less relevant. This outcome reinforces prior results showing that individuals are more willing to reduce meat consumption than to abstain from meat completely [69,70].

#### Reasons for the Preferred Option of a Dish

Furthermore, in the data analysis, great interest was placed on the reasons behind the preference for an option. Overall, *healthy* was one of the most frequently chosen reason for the no meat option. This is consistent with the fact that individuals following a plant-based diet are more health concerned, as they, for instance, were shown to drink less energy drinks and alcohol, spend more hours per week on physical activity and eat more fruits and vegetables [49].

*Right amounts* was the most frequently clicked reason for choosing the reduced meat option. Since the reduced meat version was the most chosen option in general, it can be concluded that a balanced serving style, i.e., right amounts of all food items and thus a not too crowded or messy plate, is of higher relevance. This was shown to be true prior, as Zellner and colleagues showed in 2010 that a symmetric/balanced serving style in combination with a colour was rated as more attractive than an asymmetric one [71], and in 2011, that consumers liked a balanced serving style more than for a messy one [44]. Indeed, food appearance was shown to be an important aspect in meal choice [65].

For the meaty option, the most prominent reason was *least amount of what my child does not eat*. As discussed above, this reason indicates that the child probably does not like one or more vegetables served on the plate, and since the vegetable proportions on the meaty plate were the smallest compared to the other options, the meaty dish was chosen. In fact, it cannot necessarily concluded that this is solely due to a general high affinity to meat, it could also be that the child has a dislike for vegetables presented on the plate.

### 4.5. Feasibility of Meat Reduction

As taste or distaste, familiarity, and preference for particular foods play amongst others a crucial role in the decision on what we eat [8], it is not an easy task to replace the meat-heavy diet of Germans by a more plant-based one. Another study conducted in Germany revealed that 9.5% of the examined individuals would be willing to reduce their meat consumption [51]. On the contrary, 75.1% were found to be "unconcerned meat eaters" who, if meat and sausage were cheaper, would even eat more of it [51]. However, since it was also shown that consumers (from other countries) are more willing to reduce their meat consumption than to eliminate meat completely from their diet [69,70], it is therefore of importance to investigate which paths could be taken to implement a meat-reduced diet. In addition, it is to highlight that, in contrast to the study of Cordts and colleagues from 2013, only one respondent clicked the option “*I cannot see myself reducing my meat consumption.*” in the current study. Furthermore, the preference for the reduced variant did not distinguish significantly from the meaty option in terms of parental MA, which reinforces the theory that a reduction of meat consumption is by all means feasible [69,70].

When asked for possible reasons to reduce their meat consumption, reasons related to meat production such as factory farming and animal welfare were the most chosen answer options, which contradicts the results from Cordts and colleagues from 2013, where animal welfare was the least chosen reason after environment and especially health aspects [51]. The current differences to the obtained results could have its origin in the recent increase in media coverage of scandals in meat production as “untenable conditions” in one of the largest German companies for meat production was revealed recently [72], which is only the latest discovery of a series of scandals related to meat production: German “Gammelfleischskandal” (rotten meat scandal) in 2005 [73], dioxin scandal in 2011 [74], Europe’s horse meat scandal in 2013 [75], and scandals about meat products contaminated with listeria in 2019 [76], which may have slowly manifested in people’s minds. Interestingly, the reason *medical recommendation* was clicked by less than a third of the study population; however, *health* was chosen by n = 52 participants. This might be interpreted first as contradicting and only partly consistent with Cordts and colleague’s results [51]. However, the term “health” can have many facets, starting with the personal body health, over mental health, health of all living beings, and health of planet Earth.

From the obtained results, it was further indicated that the social environment of the participant apparently had a distinguished influence, as wishes of the *partner and children* were more likely to be a reason than the fact that *colleagues and friends would avoid or reduce meat consumption*. The majority did rather disagree to the statement that they would not get any support from their social environment if they gave their child no more meat and meat products, which is contradicting with Cheah and colleagues (2020), who stated that social environment was a major barrier when individuals refuse to change their diet [77]. However, parents wrote in the open box comments about having difficulties, for example, that it is difficult to fulfil all wishes of the family, or even change something in their children’s eating habits, which hence is a hurdle in the social environment that needs to be considered when aiming for changes in children’s diet.

It was further mentioned in one comment that the respective individual would be happy if vegetarian nutrition is no longer seen so negatively by the media and the professional societies. Although the vegetarian diet was several times shown to be healthy [21,22,23,24,25,26] and in fact even healthier than a meaty diet [25,26], it is still considered as extraordinary, especially when a child abstains meat [61]. This position was strengthened by the open box comments, where many parents stated that meat is part of a balanced, healthy diet. The nutritional recommendations in the different countries include meat in a balanced diet to ensure the supply of important nutrients for growth and development [78,79]. Adjustments in the official recommendations on meat consumption and more positive media coverage of vegetarianism and veganism may be necessary to make an impact on an individual’s mindset.

### 4.6. Meat Substitutes Might Be One Solution

One path is the replacement of meat items by meat substitutes. More precisely, the meat protein is replaced by alternative proteins from plants (e.g., soy), algae (e.g., *Spirulina*), insects or by cultured meat and these alternatives receive increasing global attention [80]. However, when asked in the survey to rank four types of sauces eaten with spaghetti according to preference, it became clear that meat substitutes are not (yet) the best way to a meat-reduced diet, as here the bolognaise made from soy was voted fourth by almost half of the study population. Moreover, out of all given reasons to reduce meat consumption, those concerning optimised meat substitutes were clicked quite rarely. However, having *tastier* meat substitutes and *more choice* of meat substitutes were more important than *price* or the characteristic to *imitate meat* properly. One parent commented in an open box answer that there is no *healthy* meat substitute on the market. Another participant addressed the additives in meat substitutes, thus stressing that meat substitutes are often considered as ultra-processed food and not healthy, much less a healthy alternative to meat [81]. The results of this study are consistent with prior research, where it was shown that consumers are in fact willing to consume a more sustainable diet, but rather unwilling to consume meat substitutes [69,70,82]. Elsewhere investigated barriers for acceptance seems to be food neophobia, attitudes towards meat substitutes, unfamiliarity, lack of information, costs, and perceived lower sensory attributes in comparison to meat [82,83]. This information could be useful for public health interventions [84] and food industry to focus on the health aspect and variety when developing and producing new meat substitutes.

### 4.7. Strengths and Limitations

Qualitative interviews have been conducted beforehand to get a picture of parents’ general attitude and understanding towards children’s daily eating habits, meat consumption and meat substitutes to better design the questionnaire for this exploratory quantitative study. The online survey was an advantage for the purpose of this study as it was shown that there seems to be no significant difference in the obtained results when using picture stimuli instead of real food [39]. By hanging up posters with a QR code, parents could easily participate and take the chance to learn about nutrition and, in addition, win one out of ten children’s cooking books.

Nevertheless, the questionnaire had limitations: The assessment for meat consumption frequency and amounts are only estimates made by the participant and, compared to a diet diary in which the exact weight of each ingredient is noted, it is an imprecise measurement [46]. However, for an online survey, a diary cannot be used and a 24-h recall would be unsuitable to ask about daily meat consumption. Therefore, an estimate of the weekly meat consumption was asked through an open box answer option (instead of provided answer options), and each participant should make their own estimate. To prevent incorrect estimates, examples for the approximate weight of meat items were provided to the participant. Moreover, due to a mistake, it was missed to ask for the total number of children per respondent, as well as for the gender of the child. The latter could be of relevance when considering for example the CEBQ results, as it was shown that boys score higher on fussy eating, emotional overeating, and lower on the enjoyment of food scale than girls [37]. However, gender had not consistently an effect on eating behaviour [35,63]. Furthermore, the small sample of 90 participants resulted in low numbers of each group of MA. In addition, parents with lower education than university degree and men were relatively underrepresented in the current study. Therefore, this study only gives a small insight into the topic and should be repeated on a larger scale with more participants and a more heterogeneous study population.

### 4.8. Considerations for Future Research

To offer three variations per dish was by far not exhaustive, so parents could not choose from the provided options what they usually serve to their child. Offering more than three options would give more insights in this regards. Furthermore, even though the questionnaire emphasised meat reduction, this might not be the main aspect for an individual’s decision. In addition, the parent had to make a hypothetical choice and then answer questions on behalf of their child, which opens doors to confounders due to misinterpretations, e.g., whether a dish could be tasteful, liked and preferred over another. The presentation of a plate with three (no-meat variant) to four food items (meaty and reduced meat variant) also brings several confounders along: The personally preferred number of items, as well as the number of colours on the plate, or if the items are centred or non-centred. As an example, the no meat variant of the dish chicken drumsticks had broccoli, pumpkin seasoned with herbs, and rice with peas on the plate. Hence, one saw 2 greenish items (broccoli, rice with peas) and one orange (pumpkin) with green dots (herbs). The number of three foods should correspond to the general preference of three pieces [40,41], but the preferred number of three different colours stated in Zampollo and colleague’s studies might not have been reached, whereas the options containing chicken had an additional colour and thus changed the whole appearance of the plate. Furthermore, the arrangement of the food items on this specific plate was different compared to the other dishes, as they were arranged “in line” and the other plates were arranged more in carrés. It was shown prior that the arrangement of food items on a plate affects the preference and willingness to pay for it [85], hence, the arrangement on the plate in the current study might have had a greater influence. However, whether the serving style had a significant impact on the parents’ decision is unfortunately left unanswered in this study.

In a follow-up study, it should also be asked about the parents’ cooking skills and how much time they spend preparing meals in order to analyse the parents in more detail and to better assess the attachment to meat [66,67]. Furthermore, in order to expand the knowledge in children’s eating behaviour and also their willingness to shift from a meaty diet towards a more plant-based diet, it would be quite useful to develop a *Children’s meat attachment questionnaire* exclusively for children, as one could assess the positive bond of a child towards meat consumption and thus directly compare parental meat attachment with that of the child. Such a questionnaire should be adapted to different age groups in order to obtain reliable results for each age group. Within the framework of this study, it was not possible to develop and validate such a questionnaire; this would be a desirable future project.

## 5. Conclusions and Future Implications

The parent’s attachment to meat and associated factors such as gender, lifestyle and attitudes, influences their meal choice and therefore their child’s meat consumption and responsiveness to satiety. Parents with a higher attachment to meat and higher meat consumption chose meals with a higher meat content for their child.

The reasons associated with choosing a meaty dish for their child were first of all the parent’s MA. Out of the actively chosen reasons, tastiness, healthiness and the balanced serving style as well as habits and the child’s preferences had their fair share in the parent’s decision.

The current study reinforced that addressing the child’s social environment, such as peers and parents, is essential when it comes to changing a child’s eating behaviour [11,12,13]. By developing tastier and healthier meat substitutes and by communicating tasty vegetarian recipes, parents could become more comfortable cooking plant-based everyday meals that meet the child’s taste. In addition, the official recommendations in different countries on meat consumption should consider to adjust to today’s knowledge regarding the adequateness of a plant-based diet for children, as it was shown before [21,22,23,24,25,26], which could in the long run increase acceptability and improve the image of plant-based diets in general for individuals with high attachment to meat.

## Figures and Tables

**Table 1:** Description of the total study population and the members of the groups low, medium and high meat attachment (MA). *p*-values show if the groups distinguish, statistical significant results are marked with a star. The corresponding post hoc test (pairwise test of independence) is given by *a* (low vs. medium MA), *b* (low vs. high MA), and *c* (medium vs. high MA).

	Total	Low MA	Medium MA	High MA	*p*-Value Difference between Groups ${}^{†}$
**n (%)**	90 (100%)	22 (22.4%)	45 (50%)	23 (25.6%)	
**MA score ${}^{1}$**	15.80 ± 4.25	<13.00	13.00–18.36	>18.36	
**Gender**					
*male*	13 (14.4%)	1 (1.1%)	5 (5.6%)	7 (7.8%)	0.035 *${}^{,b}$
*female*	77 (85.6%)	21 (23.3%)	40 (44.4%)	16 (17.8%)	
**Age** **(in years)**	37.0 ± 5.2 (22–54)	36.2 ± 4.7	36.8 ± 5.9	38.1 ± 3.7	0.048 *${}^{,⊕,c}$
**Living area**					
*countryside*	14 (15.6%)	0 (0%)	6 (6.7%)	16 (17.8%)	
*suburban*	26 (28.9%)	11 (12.2%)	13 (14.4%)	21 (23.3%)	
*city*	50 (55.6%)	3 (3.3%)	7 (7.8%)	13 (14.4%)	0.106
**Education ${}^{2}$**					
*low*	5 (5.6%)	0 (0%)	4 (4.4%)	1 (1.1%)	
*moderate*	32 (35.6%)	4 (4.4%)	15 (16.7%)	13 (14.4%)	
*high*	53 (58.9%)	18 (20.0%)	26 (28.9%)	9 (10.0%)	0.035 *${}^{,b}$
**Occupation ${}^{3}$**					
*student*	8 (7.8%)	2 (2.2%)	6 (6.7%)	0 (0%)	
*worker*	73 (81.1%)	19 (21.1%)	32 (35.6%)	22 (24.4%)	
*at home*	9 (10.0%)	1 (1.1%)	7 (7.8%)	1 (1.1%)	0.129
**BMI**	23.9 ± 3.4	23.1 ± 3.4	23.6 ± 3.2	25.2 ± 3.5	
*male*	25.4 ± 2.7	22.5 ± 0.0	24.5 ± 1.6	26.5 ± 3.1	
*female*	23.6 ± 3.4	23.2 ± 3.5	23.4 ± 3.3	24.6 ± 3.6	0.071 ${}^{⊕}$
**Dietary**					
**identity**					
*omnivore*	54 (60.0%)	5 (5.6%)	28 (31.1%)	21 (23.3%)	
*flexitarian*	28 (31.1%)	11 (12.2%)	15 (16.7%)	2 (2.2%)	
*pescetarian*	2 (2.2%)	1 (1.1%)	1 (1.1%)	0 (0%)	
*vegetarian*	3 (3.3%)	2 (2.2%)	1 (1.1%)	0 (0%)	
*vegan*	3 (3.3%)	3 (3.3%)	0 (0%)	0 (0%)	<0.001 *${}^{,a,b}$
**Number of**	1.4 ± 0.6				
**children (5–8 years)**					
*1*	63 (70.0%)	13 (14.4%)	32 (25.6%)	18 (20.0%)	
*2*	23 (25.6%)	7 (7.8%)	12 (13.3%)	4 (4.4%)	
*3*	3 (3.3%)	2 (2.2%)	0 (0%)	1 (1.1%)	
*4*	1 (1.1%)	0 (0%)	1 (1.1%)	0 (0%)	0.403
**Child’s age (in years)**					
*5*	25 (27.8%)				
*6*	20 (22.2%)				
*7*	26 (28.9%)				
*8*	19 (21.1%)				

* p≤0.05. ${}^{1}$ Theoretical possible scores: 4–28. ${}^{2}$ *Low* includes no degree and secondary school, *moderate* includes A-levels and vocational training, *high* includes. University degree and PhD. ${}^{3}$ *Student* includes pupils and students, *worker* includes employee, civil servant, and self-employed persons, *at home* includes job seeker and persons on parental leave. ${}^{a}$ Low MA versus medium MA. ${}^{b}$ Low MA versus high MA. ${}^{c}$ Medium MA versus high MA. ${}^{†}$ $\chi^{2}$-test with simulated *p*-value, ${}^{⊕}$ ANOVA (instead of $\chi^{2}$-test).

**Table 2:** Frequency of meat and meat substitutes consumption, as well as average grams per week of meat consumption of the respondent and child.

		Respondent	Child
**Meat**			
Frequency	*daily*	21 (23.3%)	30 (33.3%)
	*2–3 × per week*	36 (40%)	44 (48.9%)
	*3–4 × per month*	22 (24.4%)	12 (13.3%)
	*1 × months or less*	5 (5.6%)	2 (2.2%)
	*never*	6 (6.7%)	2 (2.2%)
Grams per week:			
*Median (IQR)*		300 (157.5–600)	250 (150–500)
**Meat substitutes**			
Frequency	*daily*	1 (1.1%)	–
	*2–3 × per week*	12 (13.3%)	–
	*3–4 × per month*	17 (18.9%)	–
	*1 × months or less*	18 (20%)	–
	*never*	42 (46.7%)	–

**Table 3:** Purchasing behaviour of the total study population and the members of the groups low, medium and high meat attachment (MA) regarding meat and plant-based food. *p*-values show differences between groups, statistical significant results are marked with a star.

	Total	Low MA	Med. MA	High MA	*p*-Value
**Meat ${}^{1}$**					<0.001 *${}^{,†,a,b}$
*conventional*	8 (8.9%)	0 (0%)	3 (3.3%)	5 (5.6%)	
*organic*	36 (40.0%)	8 (8.9%)	21 (23.2%)	7 (7.8%)	
*both*	38 (42.2%)	6 (6.7%)	21 (23.2%)	11 (12.2%)	
**Plant-based** **food**					0.004 *${}^{,†,b}$
*conventional*	10 (11.1%)	0 (0%)	4 (4.4%)	6 (6.7%)	
*organic*	44 (48.9%)	14 (15.6%)	24 (26.7%)	6 (6.7%)	
*both*	36 (40.0%)	8 (8.9%)	17 (18.9%)	11 (12.2%)	

* p≤0.05. ${}^{†}$ $\chi^{2}$-test with simulated *p*-value (based on 2000 replicates). ${}^{1}$ Respondents who do not purchase meat are excluded (n = 8). ${}^{a}$ Low MA versus medium MA. ${}^{b}$ Low MA versus high MA.

## Data Availability

The authors do not have permission to make the data publicly available.

## Abbreviations

The following abbreviations are used in this manuscript:

BMI	Body mass index
CEBQ	Children behaviour questionnaire
DACH	Germany (D), Austria (A) and Switzerland (CH)
Df	Degrees of freedom
DGE	The German Nutrition Society (Deutsche Gesellschaft für Ernährung e.V.)
IQR	Interquartile range
MA	Meat attachment
MAQ	Meat attachment questionnaire
MDPI	Multidisciplinary Digital Publishing Institute
MS	Meat substitutes
